# Supplementary material for: Implementation of a Novel Case-Based Session for Medical Students Focused on Artificial Intelligence Ethics
Source: MedEdPORTAL. 2026 Jun 19;22:11611. doi: 10.15766/mep_2374-8265.11611 (PMC13279577; doi:10.15766/mep_2374-8265.11611)
Supplement: Supplementary file 1 — AI Ethics Student Guide.docxAI Ethics Facilitator Guide.docxJust-In-Time Facilitator Training Agenda.docxPre-Post Student Survey.docxLLM-Generated Summary.docx [file mep_2374-8265.11611-s001.zip › A. AI Ethics Student Guide.docx]

**Appendix A: AI Ethics Student Guide**

**Learning Objectives:**

By the end of this session, students will be able to:

1. Identify major ethical dilemmas associated with the use of AI in clinical medicine.
2. Analyze how ethical principles (e.g., autonomy, justice, beneficence) apply to AI supported clinical scenarios.
3. Evaluate potential risks and safeguards when integrating AI tools into patient care.

**Required Reading:**

- Adams, L., E. Fontaine, S. Lin, T. Crowell, V. C. H. Chung, and A. A. Gonzalez, editors. 2024. Artificial intelligence in health, health care and biomedical science: An AI code of conduct framework principles and commitments discussion draft. NAM Perspectives. Commentary, National Academy of Medicine, Washington, DC. Accessible [here](https://nam.edu/artificial-intelligence-in-health-health-care-and-biomedical-science-an-ai-code-of-conduct-principles-and-commitments-discussion-draft/)

**Recommended Resources:**

- Arora S, Jariwala SP, Balsari S. Artificial intelligence in medicine: a primer and recommendation. *J Hosp Med*. 2024; 1-4. [doi:10.1002/jhm.13371](https://doi.org/10.1002/jhm.13371)
- Nabi, J. (2018). How bioethics can shape artificial intelligence and machine learning. *Hastings Center Report*, *48*(5), 10-13. doi: [10.1002/hast.895](https://doi.org/10.1002/hast.895)
- Ethical Issues with Big Data and Artificial Intelligence. Chapter 46 In: Lo B. eds. *Resolving Ethical Dilemmas: A Guide for Clinicians, 6e*. Lippincott Williams & Wilkins, a Wolters Kluwer business; 2020. Accessible through Einstein Library.

**Session Note:**

We recognize that students bring different levels of experience and understanding to this session given how rapidly AI is evolving. Although we've prepared for this session as your course faculty, we recognize that some of you may be more knowledgeable about current AI systems and their applications, and we look forward to hearing your insights in class today. With all the technological advances and the integration of AI into our world, we are excited to discuss this important topic and learn from one another. We also welcome your feedback on this session as we strive to improve for the future.

**Suggested Session Timeline:**

- Case Discussion: AI in Medicine (35 minutes)
- Pair and Share Activity: Discuss and submit takeaway points electronically from AI curriculum (10 minutes)
- Session Wrap-up (10 minutes)
- Complete survey on AI curriculum (5 minutes)

**Case:**

A health insurance company implements a large language model (LLM) based system to assist in making coverage decisions for medical treatments. The system analyzes patient data, medical history, and proposed treatments to recommend whether a claim should be approved or denied.

A 45-year-old patient with a history of depression and anxiety is prescribed a new, expensive medication by their psychiatrist. The AI system, based on its analysis, recommends denying coverage for this medication, citing that cheaper alternatives haven't been tried first.

1. What are some reasons the AI system may not approve this care? How can we ensure that AI systems don’t become barriers to patient care?

2. How can we identify and mitigate historical biases in AI training data? What safeguards should be in place to prevent AI systems from exacerbating existing health disparities?

3. How should AI systems weigh cost considerations against individual clinical needs?

4. How can we make AI decision-making processes more transparent and interpretable for both healthcare providers and patients? How should this initial denial be explained to your patient?

5. Should there be closer human oversight of this AI-assisted decision? What do you suggest?

6. How might the implementation of AI systems to support healthcare affect the doctor-patient relationship (Consider this scenario and other contexts where the AI system supports the physician more directly).

7: As you reflect on this discussion, review the four bioethics principles (autonomy, beneficence, nonmaleficence and justice) and how each applies to integration of AI in healthcare.

**Additional Discussion Point (if time permits):**What are some ways that AI can be used to augment and improve patient care and help support physicians? On the other hand, are there other ethical dilemmas we should consider when implementing AI in these situations?

**Small Group Activity:**

[Please break into groups of 3 to 4 students. Think about today’s session and what you learned. Each group should use the QR code shared with you to submit a short summary of key points you have learned in today’s session (in 75 words or less). After class, the Course Directors will use generative AI to summarize everyone’s responses and share it back to the class via Canvas inbox message.]

*For reference – the bracketed text above is 70 words*

**During the last 5 minutes of class, please consider completing the voluntary survey to assess today’s curriculum.**
