# Supplementary material for: Implementation of a Novel Case-Based Session for Medical Students Focused on Artificial Intelligence Ethics
Source: MedEdPORTAL. 2026 Jun 19;22:11611. doi: 10.15766/mep_2374-8265.11611 (PMC13279577; doi:10.15766/mep_2374-8265.11611)
Supplement: Supplementary file 1 — AI Ethics Student Guide.docxAI Ethics Facilitator Guide.docxJust-In-Time Facilitator Training Agenda.docxPre-Post Student Survey.docxLLM-Generated Summary.docx [file mep_2374-8265.11611-s001.zip › B. AI Ethics Facilitator Guide.docx]

**Appendix B: AI Ethics Facilitator Guide**

**Learning Objectives:**

By the end of this session, students will be able to:

1. Identify major ethical dilemmas associated with the use of AI in clinical medicine.
2. Analyze how ethical principles (e.g., autonomy, justice, beneficence) apply to AI supported clinical scenarios.
3. Evaluate potential risks and safeguards when integrating AI tools into patient care.

**Required Reading:**

- Adams, L., E. Fontaine, S. Lin, T. Crowell, V. C. H. Chung, and A. A. Gonzalez, editors. 2024. Artificial intelligence in health, health care and biomedical science: An AI code of conduct framework principles and commitments discussion draft. NAM Perspectives. Commentary, National Academy of Medicine, Washington, DC. Accessible [here](https://nam.edu/artificial-intelligence-in-health-health-care-and-biomedical-science-an-ai-code-of-conduct-principles-and-commitments-discussion-draft/)

**Recommended Resources:**

- Arora S, Jariwala SP, Balsari S. Artificial intelligence in medicine: a primer and recommendation. *J Hosp Med*. 2024; 1-4. [doi:10.1002/jhm.13371](https://doi.org/10.1002/jhm.13371)
- Nabi, J. (2018). How bioethics can shape artificial intelligence and machine learning. *Hastings Center Report*, *48*(5), 10-13. doi: [10.1002/hast.895](https://doi.org/10.1002/hast.895)
- Ethical Issues with Big Data and Artificial Intelligence. Chapter 46 In: Lo B. eds. *Resolving Ethical Dilemmas: A Guide for Clinicians, 6e*. Lippincott Williams & Wilkins, a Wolters Kluwer business; 2020. Accessible through Einstein Library.

**Additional Background to help with content preparation if interested:**

- “How AI can make health better” *The Economist.* 2/15/2022. Available at [YouTube - Economist Clip](https://www.google.com/search?sca_esv=6d171d08af1b8f7f&sca_upv=1&q=AI+in+healthcare+clip&tbm=vid&source=lnms&fbs=AEQNm0Aa4sjWe7Rqy32pFwRj0UkWd8nbOJfsBGGB5IQQO6L3J5MIFhvnvU242yFxzEEp3BfRFWcyM5BvpTgNzM3vKj4s1TrkSiSdHEahe_j98B3pxgArdYwSuyfM6tTJeBCb9ad7d9I32o7CPCINZFlKRrh0caVracphap5F98RGzKWzlpUzeT2UF4ttmBm0oAKX20cE263u6fwYqhXJo9VC07bXQrysEw&sa=X&ved=2ahUKEwiU6Iqfss2IAxW4LFkFHf7GJ0kQ0pQJegQIDhAB&biw=1396&bih=663&dpr=1.38#fpstate=ive&vld=cid:83e4efe6,vid:0xSSonMIqBk,st:0)
- Weidener L, Fischer M. Proposing a Principle-Based Approach for Teaching AI Ethics in Medical Education. JMIR Med Educ 2024;10:e55368. doi: [10.2196/55368](https://doi.org/10.2196/55368)

**Session Note:**

We recognize that students bring different levels of experience and understanding to this session given how rapidly AI is evolving. Although we've prepared for this session as your course faculty, we recognize that some of you may be more knowledgeable about current AI systems and their applications, and we look forward to hearing your insights in class today. With all the technological advances and the integration of AI into our world, we are excited to discuss this important topic and learn from one another. We also welcome your feedback on this session as we strive to improve for the future.

**Suggested Session Timeline:**

- Case Discussion: AI in Medicine (35 minutes)
- Pair and Share Activity: Discuss and submit takeaway points electronically from AI curriculum (10 minutes)
- Session Wrap-up (10 minutes)
- Complete survey on AI curriculum (5 minutes)

**Case:**

A health insurance company implements a large language model (LLM) based system to assist in making coverage decisions for medical treatments. The system analyzes patient data, medical history, and proposed treatments to recommend whether a claim should be approved or denied.

A 45-year-old patient with a history of depression and anxiety is prescribed a new, expensive medication by their psychiatrist. The AI system, based on its analysis, recommends denying coverage for this medication, citing that cheaper alternatives haven't been tried first.

1. What are some reasons the AI system may not approve this care? How can we ensure that AI systems don’t become barriers to patient care?

**Faculty Notes:**

- Discuss possible biases related to development of system itself. System is only as good as how it is trained. If the AI was trained on historical data where mental health treatments were often denied or undervalued, it might perpetuate this bias in its recommendations. Biased inputs will further exacerbate discrimination.
- Another possibility is that the system may not fully understand the nuances of the patient’s condition or previous unsuccessful treatments if not recorded in the electronic medical records. Omissions and human errors in charting can perpetuate justice violations once integrated into AI systems. Students may also mention the concept of “AI hallucinations” – which describes when LLMs perceive patterns that are nonexistent and create inaccurate outputs – thereby leading to further errors by AI system decision support. This highlights the importance of human oversight in the development of these AI systems to support healthcare.
- The system might over-prioritize cost-effectiveness (i.e., recommending cheaper alternatives) over individualized patient care. Mental health medications can be highly specific to a patient's needs, and requiring cheaper alternatives could delay effective treatment, potentially worsening the patient's condition.
- It is important to realize that even with an AI system, humans that review these claims often deny them without understanding all the clinical nuances. The additional concern that occurs with AI support is that it can increase the scale of these errors dramatically.

2. How can we identify and mitigate historical biases in AI training data? What safeguards should be in place to prevent AI systems from exacerbating existing health disparities?

**Faculty Notes:**

It will be interesting to see what possible solutions students may come up with. Some suggestions may include:

- Prior to using AI systems, we should ensure that input data includes diverse patient groups and avoids over-representing certain demographics or under-representing marginalized or otherwise vulnerable populations.
- May also consider mechanisms of auditing data to make sure it is representative
- From a larger healthcare level, as these systems are being used more, you can ask students if they think there should be more regulatory oversight – for instance, institutions could have clear regulations that guide how and when patients are notified of AI support in decision-making; there can be established AI ethics committees at institutions that utilize AI (for example: hospitals, insurance companies) made up of clinicians, patients, AI experts, and ethicists. On the other hand, if there is too much regulation, institutions may fall behind with regards to AI innovation and what is offered to patients.
- Regulation could be mandated also at a state or national level. This may prove challenging given that AI use is already prevalent in similar situations, and it is challenging for regulatory bodies to keep up with the speed of innovation.

3. How should AI systems weigh cost considerations against individual clinical needs?

**Faculty Notes:**

- When using AI systems to assist in healthcare decision-making, it's crucial to balance cost considerations with individual clinical needs. Faculty should emphasize that patient care and clinical efficacy must remain the primary drivers of treatment decisions, and AI systems should be designed to prioritize patient-centered care. While cost-effectiveness is important, it should never override a patient’s specific medical requirements, particularly when cheaper alternatives may not be as effective. AI decisions must be transparent, providing clear justifications when cost is a factor, and there should be an appeal process to allow clinicians and patients to challenge decisions. Additionally, AI systems should account for both short- and long-term outcomes, recognizing that higher-cost treatments may prevent more expensive complications in the future. To prevent exacerbating health disparities, AI must be designed to ensure equitable access to necessary care, especially for vulnerable populations. Human oversight, ethical review panels, and a focus on fairness are essential safeguards in balancing cost and clinical needs.

4. How can we make AI decision-making processes more transparent and interpretable for both healthcare providers and patients? How should this initial denial be explained to your patient?

**Faculty Notes:**

- In the scenario where a health insurance company’s AI system denies coverage for a prescribed medication, transparency and the informed consent process are critical. AI systems often function as a "black box," meaning healthcare providers and patients may not fully understand how decisions are made. As AI becomes more prevalent in healthcare, it’s essential to address how informed consent can be integrated into AI-driven care. Students should spend some time generating ideas as to how to improve transparency.
- As AI becomes more integrated into healthcare, it is essential for clinicians to be transparent about its use with patients. This transparency should be part of the informed consent process, though there are challenges in educating and notifying patients about AI’s role. In situations where a health insurance company uses AI for decision support, the company should clearly inform patients. If patients are unsure of the steps to appeal a denial or how AI influences these decisions, clinicians may need to explain the process.
- Additionally, you can challenge the students to consider this on a smaller scale from the clinician’s perspective—such as how a psychiatrist using AI to suggest treatment plans would notify and involve patients in these decisions.

5. Should there be closer human oversight of this AI-assisted decision? What do you suggest?

**Faculty Notes:**

- Students may have different suggestions about appropriate human oversight.
- One possibility includes a system where the AI makes initial recommendations, but a human reviewer must approve all denials. You can ask students if they think a system like this would change outcomes and mitigate bias.

6. How might the implementation of AI systems to support healthcare affect the doctor-patient relationship (Consider this scenario and other contexts where the AI system supports the physician more directly).

**Faculty Notes:**

Students may have concerns related to the possible depersonalization of medicine based on how AI systems are implemented. The implementation of AI systems in healthcare, as seen in the initial scenario, has the potential to impact the doctor-patient relationship in significant ways. While AI can provide valuable support in decision-making, there is a concern that it could depersonalize medicine if not used thoughtfully. In their analysis students may reflect on how maintaining and even enhancing the doctor-patient relationship remains a critical aspect of professional duty, regardless of technological advancements, even as patients become more acclimated to the use of AI in medicine. The trust patients place in physicians is built on humanistic, compassionate care, where doctors not only share results and treatment options but also address patient concerns, preferences, and individual circumstances. Even as AI supports decision-making, it’s essential for physicians to remain at the center of communication, guiding patients through the process with empathy.

7. As you reflect on this discussion, review the four bioethics principles (autonomy beneficence, nonmaleficence and justice) and how each applies to integration of AI in healthcare.

**Faculty Notes:**

1. **Autonomy**: Do patients fully understand how AI is being used in their care? Informing patients appropriately is crucial, as autonomy forms the foundation of informed consent. Additionally, there are significant concerns about data privacy and how it can be maintained as technology advances.
2. **Justice**: AI in healthcare raises concerns about algorithmic bias, which may perpetuate or worsen existing health inequities. There are also issues regarding economic disparities, as access to AI systems may be limited by financial resources, potentially deepening divides within the healthcare system.
3. **Nonmaleficence**: We must minimize the risks associated with AI use, such as misdiagnosis, algorithmic biases, and privacy breaches. To mitigate these risks, institutions should implement ongoing monitoring and clear protocols for evaluating AI systems' safety and reliability.
4. **Beneficence**: AI systems should be designed with the primary goal of benefiting patients. However, since many are developed by private companies, there is a risk that they may prioritize efficiency and cost-effectiveness over patient well-being. It is critical that these systems align with the broader goals of patient care, considering both clinical outcomes and the personal circumstances of each individual.

**Additional Discussion Point (if time permits):**What are some ways that AI can be used to augment and improve patient care and help support physicians? On the other hand, are there other ethical dilemmas we should consider when implementing AI in these situations?

**Faculty Notes:**

Given how knowledgeable students are about AI, we expect students will have some examples that highlight possible uses of AI to support clinicians. If not, some examples are shown below:

- Help review patient data on a large scale, which can enhance quality assurance etc. provided that a HIPAA compliant system is in use.
- Additional check for clinical decision making: For example, can review radiology results before a clinician finalizes them
- Can help respond to patient messages via chat or email secure portals. As technology advances, could also have voice capabilities
  - For example, there are companies that have AI-based “nurses” that can call patients by phone to review basic procedures such as how to prepare for a colonoscopy
- Can dramatically help with efficiency in note-writing – using AI scribes
- Can help with our own learning: For example, at some institutions, AI systems review which patients are seen by clinical trainees, and then the AI systems automatically send learning points via email to trainees the next morning before rounds start (with notable peer-reviewed articles, etc.).
- Students may also bring up AI use to support research. Given the huge scope of today’s session, we did not focus on this but there are concerns about academic integrity. Institutional policy guiding AI use may vary. Generally, from a research perspective, AI should not be used for writing any scholarly work and most journals have guidelines that discuss AI policies (and how AI should be cited if used).

**Small Group Activity:**

[Please break into groups of 3 to 4 students. Think about today’s session and what you learned. Each group should use the QR code shared with you to submit a short summary of key points you have learned in today’s session (in 75 words or less). After class, the Course Directors will use generative AI to summarize everyone’s responses and share it back to the class via Canvas inbox message.]

*For reference – the bracketed text above is 70 words*

**During the last 5 minutes of class, please consider completing the voluntary survey to assess today’s curriculum.**
