# Supplementary material for: Implementation of a Novel Case-Based Session for Medical Students Focused on Artificial Intelligence Ethics
Source: MedEdPORTAL. 2026 Jun 19;22:11611. doi: 10.15766/mep_2374-8265.11611 (PMC13279577; doi:10.15766/mep_2374-8265.11611)
Supplement: Supplementary file 1 — AI Ethics Student Guide.docxAI Ethics Facilitator Guide.docxJust-In-Time Facilitator Training Agenda.docxPre-Post Student Survey.docxLLM-Generated Summary.docx [file mep_2374-8265.11611-s001.zip › C. Just-In-Time Facilitator Training Agenda.docx]

**Appendix C: Just-In-Time Facilitator Training Agenda**

**AI Ethics**

Course Directors welcome facilitators upon their arrival and ensure that everyone has a copy of the *Facilitator Guide for Artificial Intelligence Ethics*. It is the expectation that facilitators who participate in this session have already read through the facilitator guide and have also reviewed the pre-session materials listed in the facilitator guide (and shared via email approximately 2 weeks prior to the session).

Content from the facilitator guide is reviewed together as a group in this 45-minute training so facilitators are well prepared for the session.

Part 1. Introductions

- This training begins with very brief introductions.
- In addition to core bioethics faculty, content experts are also invited to this session to answer any nuanced questions that facilitators may have. Content experts are invited to share about their background and core bioethics faculty also introduce themselves.

Part 2. Framing of the session

- We remind the facilitators (core bioethics faculty and content experts) where the learners are in the curriculum overall.
- We review the learning objectives to ensure that we collectively understand the goals for the session.
- We also briefly go over the required and recommended resources for the session.
- Finally, we discuss small group facilitator strategies that are used throughout this course to encourage participation and a sense of belonging in this learning environment (use of ground rules, probing questions, active listening, etc.)

Part 3. Prioritize Questions and Clarify Confusion

- We begin by asking if anyone has any questions about the session content or format.
  - Course Directors answer questions related to the format and facilitation as well as how to best connect to bioethics concepts.
  - Content experts are available to answer more specific questions related to artificial intelligence.

Part 4. Review of Session Format and Content

- The overall timeline and format of the active learning session is reviewed.
- Course Directors lead discussion to review the case and each discussion question with facilitators. For each discussion question, facilitators share ideas and concepts that should be covered. Content experts also provide additional context as needed. Facilitators also use some time during the training to share potential facilitation pearls and pitfalls with each other.
- Course Directors review the end of class small group activity that asks students to submit a short summary of key points learned during the session.
- To close the session, the Course Directors thank everyone for their participation and involvement and invite feedback from facilitators to help with future iterations of this curriculum.
