# Supplementary material for: Implementation of a Novel Case-Based Session for Medical Students Focused on Artificial Intelligence Ethics
Source: MedEdPORTAL. 2026 Jun 19;22:11611. doi: 10.15766/mep_2374-8265.11611 (PMC13279577; doi:10.15766/mep_2374-8265.11611)
Supplement: Supplementary file 1 — AI Ethics Student Guide.docxAI Ethics Facilitator Guide.docxJust-In-Time Facilitator Training Agenda.docxPre-Post Student Survey.docxLLM-Generated Summary.docx [file mep_2374-8265.11611-s001.zip › D. Pre-Post Student Survey.docx]

**Appendix D: Pre-Post Student Survey**

Please rate your ability to do each of the following after participating in the Artificial Intelligence (AI) Ethics session. Your responses are anonymous.

1. **I can identify how the principle of autonomy applies when using AI in clinical care.**

|  |  | 1 = strongly disagree | 2 = disagree | 3 = neutral | 4 = agree | 5 = strongly agree |
| --- | --- | --- | --- | --- | --- | --- |
| BEFORE: |  |  |  |  |  |  |
| AFTER: |  |  |  |  |  |  |

1. **I can identify how the principle of beneficence applies when using AI in clinical care.**

|  | 1 = strongly disagree | 2 = disagree | 3 = neutral | 4 = agree | 5 = strongly agree |
| --- | --- | --- | --- | --- | --- |
| BEFORE: |  |  |  |  |  |
| AFTER: |  |  |  |  |  |

1. **I can identify how the principle of nonmaleficence applies when using AI in clinical care.**

|  | 1 = strongly disagree | 2 = disagree | 3 = neutral | 4 = agree | 5 = strongly agree |
| --- | --- | --- | --- | --- | --- |
| BEFORE: |  |  |  |  |  |
| AFTER: |  |  |  |  |  |

1. **I can identify how the principle of justice applies when using AI in clinical care.**

|  | 1 = strongly disagree | 2 = disagree | 3 = neutral | 4 = agree | 5 = strongly agree |
| --- | --- | --- | --- | --- | --- |
| BEFORE: |  |  |  |  |  |
| AFTER: |  |  |  |  |  |

**5. I can identify ethical concerns related to bias and inequity when using AI in clinical care.**

|  | 1 = strongly disagree | 2 = disagree | 3 = neutral | 4 = agree | 5 = strongly agree |
| --- | --- | --- | --- | --- | --- |
| BEFORE: |  |  |  |  |  |
| AFTER: |  |  |  |  |  |

**6. I can identify ethical concerns related to patient privacy when using AI in clinical care.**

|  | 1 = strongly disagree | 2 = disagree | 3 = neutral | 4 = agree | 5 = strongly agree |
| --- | --- | --- | --- | --- | --- |
| BEFORE: |  |  |  |  |  |
| AFTER: |  |  |  |  |  |

**7. I can identify ethical concerns related to potential harm when using AI in clinical care.**

|  | 1 = strongly disagree | 2 = disagree | 3 = neutral | 4 = agree | 5 = strongly agree |
| --- | --- | --- | --- | --- | --- |
| BEFORE: |  |  |  |  |  |
| AFTER: |  |  |  |  |  |

**8. This session was well-run.**

1. = strongly disagree 2 = disagree 3 = neutral 4 = agree 5 = strongly agree

**9. This session was engaging.**

1. = strongly disagree 2 = disagree 3 = neutral 4 = agree 5 = strongly agree

**10. This session was relevant.**

1. = strongly disagree 2 = disagree 3 = neutral 4 = agree 5 = strongly agree

**11. This session was timely.**

1. = strongly disagree 2 = disagree 3 = neutral 4 = agree 5 = strongly agree

**12. This session should be included in the bioethics pre-clerkship course in the future.**

1. = strongly disagree 2 = disagree 3 = neutral 4 = agree 5 = strongly agree

**Plus-Delta Comments (adapted from Helminski and Roth)^^[[1]](#footnote-1)^,^[[2]](#footnote-2)^^:**

**+** What aspects of this session were especially useful, valuable, interesting, or new?

**Δ** If this session were repeated, what recommendations do you have to improve learning?

1. Helminski, L. & Koberna, S. (1995). Total quality in instruction: A systems approach. In H. V. Roberts (Ed.), Academic initiatives in total quality for higher education (pp309-362). Milwaukee, WI: ASQC Quality Press [↑](#footnote-ref-1)
2. Roth LT, Friedman S, Gordon R, Catallozzi M. Rainbows and “Ready for Residency”: Integrating LGBTQ Health Into Medical Education. MedEdPORTAL. 2020;16:11013. <https://doi.org/10.15766/mep_2374-8265.11013> [↑](#footnote-ref-2)
