# Supplementary material for: Implementation of a Novel Case-Based Session for Medical Students Focused on Artificial Intelligence Ethics
Source: MedEdPORTAL. 2026 Jun 19;22:11611. doi: 10.15766/mep_2374-8265.11611 (PMC13279577; doi:10.15766/mep_2374-8265.11611)
Supplement: Supplementary file 1 — AI Ethics Student Guide.docxAI Ethics Facilitator Guide.docxJust-In-Time Facilitator Training Agenda.docxPre-Post Student Survey.docxLLM-Generated Summary.docx [file mep_2374-8265.11611-s001.zip › E. LLM-Generated Summary.docx]

**Appendix E: LLM-Generated Summary**

**ChatGPT‑4o ^[[1]](#footnote-1)^-generated summary of student responses from small group activity at the end of session where students were asked to work together in groups of 3 to 4 students and submit what they learned:**

"In this session, we discussed the potential benefits and challenges of using AI in healthcare. Key advantages include increased efficiency, cost savings, and alleviating physician workload, particularly in administrative tasks like scribing. However, there are significant concerns, such as biases in AI models, lack of transparency (the "black box" phenomenon), and potential impacts on patient autonomy, confidentiality, and fairness.

We emphasized the importance of human oversight in AI's use, especially in clinical decision-making, as AI can make mistakes or perpetuate existing biases. AI models must be trained on diverse, representative data to avoid bias and ensure equitable outcomes across different populations. Additionally, AI cannot replace human judgment, particularly in areas like diagnosis or treatment decisions, as it lacks the ability to account for the full context of patient care.

The discussion highlighted the ethical concerns of using AI in medicine, such as ensuring informed consent, maintaining patient privacy, and preventing the misuse of AI for profit (e.g., by insurance companies). Clear regulations and external oversight are necessary to prevent AI from reinforcing healthcare disparities or making harmful decisions. It was concluded that while AI has great potential in healthcare, its use must be carefully managed with continuous monitoring, transparency, and validation by trained professionals to safeguard patient welfare and ensure ethical application.”

1. OpenAI. ChatGPT. Large language model. <https://chat.openai.com> [↑](#footnote-ref-1)
