# Supplementary material for: Implementation of a Novel Case-Based Session for Medical Students Focused on Artificial Intelligence Ethics
Source: MedEdPORTAL. 2026 Jun 19;22:11611. doi: 10.15766/mep_2374-8265.11611 (PMC13279577; doi:10.15766/mep_2374-8265.11611)
Supplement: Supplementary file 1 — AI Ethics Student Guide.docxAI Ethics Facilitator Guide.docxJust-In-Time Facilitator Training Agenda.docxPre-Post Student Survey.docxLLM-Generated Summary.docx [file mep_2374-8265.11611-s001.zip › _Educational Summary Report_11611.pdf]

MedEdPORTAL
THE AAMC JOURNAL OF TEACHING AND LEARNING RESOURCES

Original Publication

Open Access

Implementation of a Novel Case-Based Session for Medical Students Focused on Artificial Intelligence Ethics

Danielle M. Fernandes, MD*, Talya Lisker, MS, Shitij Arora, MBBS, Aaron Hui, Adira Hulkower, JD, MS, HEC-C, Sunit Jariwala, MD, Janice Thomas John, DO, MS, MPH

*Corresponding author: dfernandes@Montefiore.org

Abstract

Introduction: As artificial intelligence (AI) is integrated into health care, it is critical for physicians to understand the ethical foundations of its use in medicine so that they can provide just care to patients and use AI technology to effectively support clinical care. This novel ethics session was designed to provide students with the opportunity to discuss ethical principles related to the use of AI in medicine. **Methods:** We designed a case-based small-group session for preclerkship medical students as part of their required bioethics course. Interdisciplinary bioethics faculty facilitated this session. After the session, participants completed a retrospective pre-post survey with questions on a 5-point Likert scale and open-ended questions. **Results:** One hundred seventy students attended the session, and 94 completed the survey (response rate 55%). Students reported a stronger understanding of the ethical issues surrounding AI use in medicine following the session. Content analysis of narrative responses showed that students valued the opportunity to discuss AI ethics with peers and facilitators. **Discussion:** Students valued this innovative session and recommended it be repeated in future years. Data from this session demonstrate a self-reported improvement in understanding of core bioethics concepts related to AI use in medicine. This case-based small-group session offers a timely and effective approach for integrating core domains of AI ethics—bias and inequity, data privacy and patient autonomy, and potential harms of AI—into the undergraduate medical school education curriculum, providing students with a foundational understanding as they prepare to use AI throughout their careers.

Keywords
Artificial Intelligence, Ethics/Bioethics, Case-Based Learning

Educational Objectives

By the end of this activity, learners will be able to:

1. Identify major ethical dilemmas associated with the use of artificial intelligence (AI) in clinical medicine.
2. Analyze how ethical principles (eg, autonomy, beneficence, nonmaleficence, justice) apply to AI supported clinical scenarios.
3. Evaluate potential risks and safeguards when integrating AI tools into patient care.

Introduction

In the past few years, artificial intelligence (AI) has become embedded in clinical care to support clinicians and deliver efficient, effective patient care. With these advances, it is vital for clinicians to understand the bioethical foundations of AI use. Ethical questions related to bias and appropriate training of AI systems, data privacy, patient autonomy, and possible harms associated with AI integration are essential for clinicians to understand so that they are better prepared to work with AI and navigate its ethical use throughout their careers. If there is bias related to the training of AI systems and the integration of AI in health care, there will be increased inequity in the clinical care environment, which in turn makes it harder to provide just care. Previous researchers have highlighted the importance of integrating AI teaching into medical education, but published curricula focused on structured AI ethics instruction are limited.[1-5] Although there is 1 curriculum by Agarwal et al. in *MedEdPORTAL* that reviews basics of AI tools through an online module for medical students, there are limited curricula available focused on AI ethics education.[6] In particular, there are few published curricula incorporating case-based small-group sessions, even though there is significant interest among students and faculty in furthering AI education in medical school.[6-11] Of note, Agarwal

Citation:
Fernandes DM, Lisker T, Arora S, et al. Implementation of a novel case-based session for medical students focused on artificial intelligence ethics. *MedEdPORTAL*. 2026;22:11611.
https://doi.org/10.15766/mep_2374-8265.11611

Copyright © 2026 Fernandes et al. This is an open-access publication distributed under the terms of the Creative Commons Attribution license.

et al. suggest that future related curricula should provide education in AI ethics, given student feedback from their study. In a recent scoping review on AI ethics education, Weidener and Fischer also further recommended that AI ethics curricula use case-based learning with real-world examples to teach students.[10] In developing this session, we recognized the importance of strengthening students' understanding of the ethical foundations of appropriate AI use in medicine. We also aimed to address the need for a timely curriculum given the rapid integration of AI into clinical practice in the past few years. We created a case-based small-group session centered on AI ethics and included it as part of our preclerkship bioethics course for all second-year medical students. This session is innovative in its use of case-based discussion to apply the 4 basic principles of bioethics—autonomy, beneficence, nonmaleficence, and justice—to the ethical challenges of AI use in clinical care.

Methods

Design

We designed this session using Kern's 6-step approach to curriculum development.[12] We conducted a needs assessment to gather information about students' prior knowledge and interest in particular AI ethics subject matter. We used information from this needs assessment to help design this session, which is a component of our bioethics course for preclerkship second-year medical students. In designing this session, we chose a case-based approach because it has demonstrated effectiveness in teaching ethics in medical education settings to students and residents.[13,14] Students applied foundational concepts of autonomy, beneficence, nonmaleficence, and justice as they relate to AI use described in the case scenario. Our overarching medical education study aims were to explore satisfaction and reaction of students to this session and to assess self-reported learning of ethical concepts in alignment with the session's learning objectives. Our session learning objectives were to:

1. Identify major ethical dilemmas associated with the use of AI in clinical medicine.
2. Analyze how ethical principles (eg, autonomy, justice, beneficence) apply to AI supported clinical scenarios.
3. Evaluate potential risks and safeguards when integrating AI tools into patient care.

The session was designed by an interdisciplinary team of bioethics faculty, medical education faculty, and AI content experts and was delivered and formally studied in 2024. This medical education study was reviewed by Albert Einstein College of Medicine's Institutional Review Board and determined to be exempt (No. 2024–16328).

Session Structure

Prior to the session, we assigned students a short reading from the National Academy of Medicine describing a proposed "Code of Conduct" framework for responsibly integrating AI in health care.[15] We also provided additional recommended resources to students and facilitators to give further background on AI use in medicine and the major tenets of AI ethics (Appendices A and B). Students were expected to spend approximately 1 hour of time preparing for this session.

A 45-minute just-in-time facilitator training, led by AI content experts on the day of the session, reviewed background information and the major ethical concepts to be discussed, and provided facilitators with additional guidance on leading case-based conversations about AI in clinical care. More information about this training is provided in Appendix C. After the facilitator training, an optional 45-minute lecture was delivered by 3 AI content experts to introduce foundational concepts in AI and describe how AI is currently used at our institution. The lecture was optional for both students and facilitators. Lecture slides were made available to individuals who did not attend to ensure consistent access to the background information.

Following this optional lecture, students and facilitators attended the required 60-minute case-based small-group discussion session, during which they critically reviewed a clinical scenario and related questions with their peers and facilitators. Small-group discussions were led by trained facilitators, some of whom designed the session and others who were distinct from the interdisciplinary team of bioethics faculty, medical education specialists, and AI content experts who designed the session. These small-group discussion sessions were held in person in different classrooms concurrently and were facilitated by our bioethics faculty. Each small group was facilitated by 1 or 2 faculty members, depending on facilitator availability and preference for co-facilitation The total class of 170 second-year students was randomly divided into 13 small-group sessions, with approximately 13 students per group. We used small-group sessions so that students could engage in discussions with peers with facilitator support. The small-group sessions were designed to be delivered independently of the lecture and had unique session learning objectives, as described earlier. The details of the case-based small-group session are further described in the student and facilitator guides (see Appendices A and B). An agenda detailing the training session is provided in Appendix C. The case included in the discussion guide describes a health insurance company that is now using a large language model (LLM) to assist in timely medication authorization decisions for

Copyright © 2026 Fernandes et al. This is an open-access publication distributed under the terms of the Creative Commons Attribution license.

patient prescriptions. The scenario then describes a patient with depression and anxiety who is prescribed a medication that is denied by this AI system because the system cites other, more cost-effective alternatives that have not been tried. Discussion questions focused on how AI systems are trained, how to protect against bias, how AI can balance costs with patient needs, and overall transparency regarding AI use. Later questions explored AI's effects on the doctor–patient relationship. Students were then explicitly asked to demonstrate, through discussion with peers, how each of the 4 bioethics principles (autonomy, beneficence, nonmaleficence, and justice) applies to the integration of AI in health care, with facilitators providing feedback to help ensure the session's learning objectives were achieved. Students were asked to discuss ways AI can improve patient care and other ethical dilemmas to be considered during the implementation of AI systems.

At the end of the discussion session, students worked with 2 or 3 peers and submitted a short description of what they learned using a QR code link provided in the student guide. These student responses were then entered into ChatGPT-4o, which generated a short summary of the responses. Several days after the course session, this LLM-generated summary was shared with the students via email. We incorporated this innovative generative AI–produced summary to demonstrate real-time AI use and efficiently share synthesized responses with students. This feature was included as part of the instructional design but was not formally evaluated.

Session Evaluation

Following the session, all students were invited to evaluate it through an anonymous online survey administered via the Qualtrics platform. The evaluation measured students' satisfaction and reactions to this bioethics course session, and assessed self-reported learning of ethical concepts using a retrospective pre-post survey. Although the case-based session was mandatory for students, completing this survey was optional. Similar pre-post retrospective surveys have demonstrated validity in previous curriculum assessment research.[16,17] A retrospective pre-post survey was used to mitigate response shift bias and reduce survey burden on our otherwise very busy target population of medical students.[16-18] The survey was adapted from a similar evaluation tool that has been previously studied and adapted to this session's goals and objectives.[19] The survey was reviewed and piloted by medical students who have previously taken the course and facilitators who are not primary investigators to establish clarity of questions. After pilot testing, minor revisions were made to the survey to integrate student and facilitator feedback. No formal validity testing was performed. The survey consisted of questions on a 5-point Likert scale to evaluate perceived learning of goals and objectives and 2 qualitative feedback questions to help inform future sessions (Appendix D). The questions that used a 5-point Likert scale focused on self-reported learning of the specific ethical concepts that were discussed and asked students to rate whether the session was well run, engaging, relevant, timely, and should be included in the course in the future. Finally, 2 qualitative questions were included to help us understand what was useful and valuable to students and learn how the session could be enhanced. All students enrolled in the course were invited to participate in this optional survey via email. A QR code was also posted in each classroom that linked to the survey anonymously. The last 5 minutes of the session were allotted for students to complete this optional survey. To incentivize participation, 10 students who participated were randomly selected to receive a $10 Amazon gift card.

Analysis

Survey results were analyzed using paired *t* tests for survey questions 1-7 and descriptive summary statistics for questions 8-12. Qualitative data from the last 2 survey questions were evaluated using content analysis. Two researchers independently analyzed the responses to open-ended questions to identify recurring themes. After the 2 researchers independently identified themes, they met to develop a coding framework with a list of agreed-upon themes. The researchers defined each of the themes together and then coded the responses independently. Discrepancies in coding were resolved through discussion between the 2 researchers. This qualitative analysis methodology was adapted from previous research.[20]

Results

This session was conducted in the fall of 2024. One hundred seventy students participated in the 13 small-group discussion sessions. Of the 22 small-group facilitators, 12 were physicians and 10 were from interdisciplinary backgrounds, including law and nursing, all with prior bioethics facilitation experience. Ninety-four students responded to the pre-post survey (response rate 55.3%). The average scores for the self-reported content retention questions were computed and are shown in Table 1. Students reported an improvement in their understanding of the ethical concepts covered in this session across all domains following the session (see Table 1). They reported a better understanding of ethical dilemmas related to AI use regarding patient autonomy, as well as the principles of beneficence, nonmaleficence, and justice in clinical care. They also reported

Copyright © 2026 Fernandes et al. This is an open-access publication distributed under the terms of the Creative Commons Attribution license.

Table 1. Results of Retrospective Pre-Post Evaluations From Students (*N* = 94)

Question	Pre *M*[a]	Post *M*[a]	*P*
I can identify how the principle of autonomy applies when using AI in clinical care.	3.39	4.23	<.001
I can identify how the principle of beneficence applies when using AI in clinical care.	3.47	4.34	<.001
I can identify how the principle of nonmaleficence applies when using AI in clinical care.	3.50	4.30	<.001
I can identify how the principle of justice applies when using AI in clinical care.	3.39	4.43	<.001
I can identify ethical concerns related to bias and inequity when using AI in clinical care.	3.60	4.56	<.001
I can identify ethical concerns related to patient privacy when using AI in clinical care.	3.71	4.43	<.001
I can identify ethical concerns related to potential harm when using AI in clinical care.	3.77	4.57	<.001

[a]Rated on a 5-point scale (1 = *strongly disagree*, 5 = *strongly agree*).

a better understanding of ethical concerns related to bias and equity, patient privacy, and potential harms of AI use in clinical health care. Students were asked about the effectiveness of the session overall, as shown in Table 2. The mean score for execution and planning was 4.29 out of 5, the mean score for engagement was 4.19 out of 5, the mean score for relevance was 4.51 out of 5, and the mean score for timeliness was 4.34 out of 5. When asked whether the session should be included in the bioethics course moving forward, the mean response was 4.36 out of 5, with 1 student noting that “it is an essential class to have now as a part of our curriculum.”

Toward the end of the session, students worked with 2-3 peers and collaboratively submitted a few sentences, highlighting what they learned. The course directors then compiled the 55 responses that were submitted and used ChatGPT-4o to produce a 3-paragraph summary of what all the students learned. This was shared with students and facilitators via email after the session to help synthesize learning. The generative AI summary is shown in Appendix E. In this summary, students reported a deeper understanding of the benefits and harms of using AI in health care and acknowledged major concerns about equity and the potential to perpetuate bias. Students also acknowledged the challenges with informed consent and patient privacy and discussed the need for better regulation overall.

There were also 86 responses to the open-ended questions that asked students to describe what was valuable and what could be improved. Among the topics considered most valuable, key themes were identified, among which appreciation for small groups and discussion, and an understanding of the use of AI in health care, were the most cited (Table 3). Students also commented on the timeliness and relevance of the session discussing AI ethics in health care. One student commented that “the information presented was very current and discussed how AI can be implemented today and how it is currently…evolving.” Students cited a desire for overall adjustment to the cases presented in the session, as well as having more time for discussion (Table 4). Of note, 21 students reported that they would not make any changes to the session. Representative quotes for each theme are listed in Tables 3 and 4. Some students also remarked on the importance of discussing AI in health care, as “these are very relevant concepts and physicians need to be trained on how to navigate difficult ethical scenarios about these concepts.” One student specifically commented on AI’s effect on the doctor–patient relationship and stated that they “didn’t necessarily realize how there may be downstream effects of how AI could affect that relationship and trust. This is a very complicated issue, and it was great to hear both sides of the topic.” Overall, students highlighted their appreciation for this introduction to basic AI concepts with 1 student summarizing that “it was very useful to discuss the concepts of AI integration in health care decision making, insurance policy decisions, and overreliance. These are very relevant concepts and physicians need to be trained on how to navigate difficult ethical scenarios about these concepts.”

Table 2. Mean Quantitative Scores From Student Evaluations (*N* = 94)

Item	Score[a]
This session was well-run.	4.29
This session was engaging.	4.19
This session was relevant.	4.51
This session was timely.	4.34
This session should be included in the bioethics preclerkship course in the future.	4.36

[a]Rated on a 5-point scale (1 = *strongly disagree*, 5 = *strongly agree*).

Discussion

As AI use becomes pervasive in health care, AI education is critical for medical students. In the past few years, proposed frameworks for teaching AI have emphasized curricula focused on the ethical use of AI, equipping medical students to better use this technology to support their clinical practice.[9,21,22] This important session addresses this need and curricular gap while giving students a foundational understanding of core bioethics principles that are essential for clinicians to understand, such

Copyright © 2026 Fernandes et al. This is an open-access publication distributed under the terms of the Creative Commons Attribution license.

MedEdPORTAL
THE AAMC JOURNAL OF TEACHING AND LEARNING RESOURCES

Table 3. Aspects of the Session That Were Valuable, Interesting, or New (*N* = 86)

Theme	No.	Representative Quotes
AI use in clinical care	17	"Discussing AI in health care is a very relevant topic to the world we live in now. I find it useful to hear how AI can be used ethically to advance the health of the population and increase efficiency."
Discussion space/session structure	16	"Open discussion on this topic, and any others in bioethics, is one of the most important aspects of the session, as it allows for a variety of viewpoints to be shared and discussed without restriction from specific prompts."
General comments related to session	12	"In my opinion all aspects and content discussed was useful, valuable, interesting, and new. I've never had formal instruction on AI and health care/medicine. Taking the approach of bioethical concerns was especially interesting."
Discussing AI ethics	11	"Hearing a wide range of perspectives on complex ethical issues relating to AI benefits us by promoting a more considerate group of medical professionals who will now be better prepared to contribute to these issues as they develop in health care."
Learning from peers	6	"Also the different backgrounds students brought to the discussion - interesting to hear from peers who have experience working on AI (ie during gap years before med school)."

Abbreviation: AI, artificial intelligence.

as data privacy, bias and equity concerns, and potential harms related to AI integration in health care. This focus on AI ethics is a major strength of our session. In contrast to other curricula that highlight technological advances in AI, our session centers on the ethical dimensions of AI as it is integrated into health care and gives students an opportunity to critically examine issues such as human oversight and the preservation of core professional values. Given the rapid pace of AI change, we intentionally focused this session on broad, foundational concepts related to AI ethics. We believe that although AI support systems are changing at a rapid pace, the overarching ethical concepts that we cover in this session, related to privacy, autonomy, and bias, will likely remain relevant in the years ahead.

In our results, students reported achieving the session's learning objectives and indicated that their self-reported understanding of the ethical implications of AI use in health care improved following the session. Student feedback also demonstrated that they valued this learning opportunity overall, with the majority recommending that this session be included in the curriculum moving forward. With its case-based discussion format, students worked with peers to identify ethical issues related to the use of AI in medicine and to demonstrate how the core bioethics principles apply in clinical scenarios.

Another major strength of our session is that it emphasizes the importance of the doctor–patient relationship and highlights the human role that physicians provide. The discussion questions empower students to consider the role of human oversight as AI is integrated in health care and discuss AI's effects on the doctor–patient relationship. Although AI has the power to dramatically change how medicine is practiced, researchers have commented on the importance of physicians preserving the "art of caring" and human connection in clinical care.[23-25] It is essential to preserve core humanistic values—such as empathy and compassion—in the doctor–patient relationship, and to explicitly emphasize these principles in educational sessions on AI for medical students.

Another strength of this session is its feasibility to be replicated at other institutions given that it was successfully delivered by facilitators who did not have significant background knowledge about AI use in clinical care. Although the discussion guide was developed by content experts, most small-group sessions were led by facilitators without specialized training. To prepare facilitators, we held a just-in-time training session with our content experts and reviewed the discussion guide and pertinent concepts to cover. With this preparation, facilitators successfully delivered the session, as evidenced by the positive responses

Table 4. Recommendations to Improve Learning in Future Sessions (*N* = 86)

Theme	No.	Representative Quotes
General suggestions about clinical scenario prompt	14	"More cases/real-life applications."<br>"While I appreciated the open-endedness of the prompt, I think some of the best discussions I've been a part of in this course have come from there being a very interesting case with a lot of nuances."
AI use in medicine/clinical practice	13	"It could be useful to include information/facts about how AI is currently being used in medicine, as opposed to solely speaking about theoretical situations."<br>"Include more discussion of things to avoid when using AI and ways it may be harmful."
Session scheduling and overall structure	11	"I think it should be more than 1 session. It's a very involved topic and is only becoming more relevant."
Less focus on insurance in the case scenario	9	"Should include more scenarios about how AI can affect medicine outside of insurance."
More focus on basic ethics	7	"I think having more of the questions focused on ethical principles would be more helpful during the small group discussion!"

Abbreviation: AI, artificial intelligence.

Copyright © 2026 Fernandes et al. This is an open-access publication distributed under the terms of the Creative Commons Attribution license.

in our student surveys. We believe that this session can be widely disseminated and adapted for use other institutions, which is especially important given that few facilitators may have background knowledge on AI ethics.

A primary limitation of this study is that it measures level 1 and level 2 learning by the Kirkpatrick Model and asks students for their reactions and self-reported learning of major ethical concepts and learning objectives.[26] As we further develop AI curricula, we plan to assess understanding of these concepts at a higher level more longitudinally and use more objective measures of learning, such as simulation-based assessments. Future iterations can also consider expanding to other ethics frameworks beyond what was described in this session.

Another limitation is that the survey response rate of 55% may affect the generalizability of the study results and introduce self-selection bias among students who chose to complete the survey. Although a higher response rate would have been better, a meta-analysis by Wu et al.[27] supports that average rates for educational online survey research may be closer to 44%, which suggests that this response rate is adequate, especially for a busy target population such as medical students. The retrospective format of this pre-post survey may also introduce recall bias, but it was chosen to minimize survey burden on students and to help minimize response shift bias.[16-18]

Despite these limitations, this session was very well received by students and addressed a key gap in their medical education. We look forward to building on this session in the future. Although sessions on AI may be challenging to teach given the dynamic nature of AI technology, this session is invaluable because it highlights foundational concepts of AI ethics that are timely and relevant in the current medical education environment.

Appendices

A. AI Ethics Student Guide.docx

B. AI Ethics Facilitator Guide.docx

C. Just-In-Time Facilitator Training Agenda.docx

D. Pre-Post Student Survey.docx

E. LLM-Generated Summary.docx

All appendices are peer reviewed as integral parts of the Original Publication.

Danielle M. Fernandes, MD: Assistant Professor, Department of Pediatrics, Albert Einstein College of Medicine; ORCID: https://orcid.org/0000-0001-9196-4777

Talya Lisker, MS: Medical Student, Albert Einstein College of Medicine

Shitij Arora, MBBS: Professor, Department of Medicine, Albert Einstein College of Medicine

Aaron Hui: Medical Student, Albert Einstein College of Medicine

Adira Hulkower, JD, MS, HEC-C: Assistant Professor, Department of Epidemiology and Population Health, Albert Einstein College of Medicine

Sunit Jariwala, MD: Professor, Department of Medicine, Albert Einstein College of Medicine

Janice Thomas John, DO, MS, MPH: Associate Professor, Department of Pediatrics, Albert Einstein College of Medicine

Disclosures
None to report.

Funding/Support
Dr. Danielle M. Fernandes received funding support from the Albert Einstein College of Medicine.

Ethical Approval
The Albert Einstein College of Medicine Institutional Review Board reviewed this project.

References

1. Masters K. Artificial intelligence in medical education. *Med Teach*. 2019;41(9):976-980. https://doi.org/10.1080/0142159X.2019.1595557
2. Srivastava TK, Waghmare L. Implications of artificial intelligence (AI) on dynamics of medical education and care: a perspective. *J Clin Diagn Res*. 2020;14(03):JI01-JI02. https://doi.org/10.7860/JCDR/2020/43293.13565
3. Park SH, Do KH, Kim S, Park JH, Lim YS. What should medical students know about artificial intelligence in medicine? *J Educ Eval Health Prof*. 2019;16:18. https://doi.org/10.3352/jeehp.2019.16.18
4. Lee J, Wu AS, Li D, Kulasegaram KM. Artificial intelligence in undergraduate medical education: a scoping review. *Acad Med*. 2021;96(suppl 2):62-70. https://doi.org/10.1097/ACM.0000000000004291
5. Farmakidis AL, Singh A, Leaf K, Rossi A. Artificial intelligence curricula in U.S. and Canadian medical schools. Data Snapshot. AAMC; 2025. https://www.aamc.org/media/84666/download
6. Agarwal G, Ramamoorthi L, Yuen T, et al. Exploring applications of artificial intelligence tools in clinical care and health professions education: an online module for students. *MedEdPORTAL*. 2025;21:11524. https://doi.org/10.15766/mep_2374-8265.11524

Copyright © 2026 Fernandes et al. This is an open-access publication distributed under the terms of the Creative Commons Attribution license.

7. Abid A, Murugan A, Banerjee I, Purkayastha S, Trivedi H, Gichoya J. AI education for fourth-year medical students: two-year experience of a web-based, self-guided curriculum and mixed methods study. *JMIR Med Educ*. 2024;10:e46500. https://doi.org/10.2196/46500

8. Weidener L, Fischer M. Artificial intelligence in medicine: cross-sectional study among medical students on application, education, and ethical aspects. *JMIR Med Educ*. 2024;10:e51247. https://doi.org/10.2196/51247

9. Tolentino R, Baradaran A, Gore G, Pluye P, Abbasgholizadeh-Rahimi S. Curriculum frameworks and educational programs in AI for medical students, residents, and practicing physicians: scoping review. *JMIR Med Educ*. 2024;10:e54793. https://doi.org/10.2196/54793

10. Weidener L, Fischer M. Teaching AI ethics in medical education: a scoping review of current literature and practices. *Perspect Med Educ*. 2023;12(1):399-410. https://doi.org/10.5334/pme.954

11. Blanco MA, Nelson SW, Ramesh S, et al. Integrating artificial intelligence into medical education: a roadmap informed by a survey of faculty and students. *Med Educ Online*. 2025;30(1): 2531177. https://doi.org/10.1080/10872981.2025.2531177

12. Thomas PA, Kern DE, Hughes MT, Tackett SA, Chen BY, eds. *Curriculum Development for Medical Education: A Six-Step Approach*. Johns Hopkins University Press; 2022.

13. Souza AD, Vaswani V. Diversity in approach to teaching and assessing ethics education for medical undergraduates: a scoping review. *Ann Med Surg (Lond)*. 2020;56:178-185. https://doi.org/10.1016/j.amsu.2020.06.028

14. Jensen NC, Cocks M, Brintz BJ, Stoff B, Cipriano SD. Structured case-based ethics discussion for trainees and faculty on dermatopathology. *MedEdPORTAL*. 2023;19:11314. https://doi.org/10.15766/mep_2374-8265.11314

15. Adams L, Fontaine E, Lin S, Crowell T, Chung VCH, Gonazalez AA, eds. Artificial intelligence in health, health care and biomedical science: an AI code of conduct framework principles and commitments discussion draft. *NAM Perspectives*. Commentary. National Academy of Medicine; 2024. https://doi.org/10.31478/202109b

16. Bhanji F, Gottesman R, de Grave W, Steinert Y, Winer LR. The retrospective pre-post: a practical method to evaluate learning from an educational program. *Acad Emerg Med*. 2012;19(2): 189-194. https://doi.org/10.1111/j.1553-2712.2011.01270.x

17. Little TD, Chang R, Gorrall BK, et al. The retrospective pretest–posttest design redux: on its validity as an alternative to traditional pretest–posttest measurement. *Int J Behav Dev*. 2020; 44(2):175-183. https://doi.org/10.1177/0165025419877973

18. Thomas EV, Wells R, Baumann SD, et al. Comparing traditional versus retrospective pre-/post-assessment in an interdisciplinary leadership training program. *Matern Child Health J*. 2019;23(2): 191-200. https://doi.org/10.1007/s10995-018-2615-x

19. Roth LT, Friedman S, Gordon R, Catallozzi M, Rainbows and “Ready for Residency”: integrating LGBTQ health into medical education. *MedEdPORTAL*. 2020;16:11013. https://doi.org/10.15766/mep_2374-8265.11013

20. Hsieh HF, Shannon SE. Three approaches to qualitative content analysis. *Qual Health Res*. 2005;15(9):1277-1288. https://doi.org/10.1177/1049732305276687

21. Paranjape K, Schinkel M, Nannan Panday R, Car J, Nanayakkara P. Introducing artificial intelligence training in medical education. *JMIR Med Educ*. 2019;5(2):e16048. https://doi.org/10.2196/16048

22. Triola MM, Rodman A. Integrating generative artificial intelligence into medical education: curriculum, policy, and governance strategies. *Acad Med*. 2025;100(4):413-418. https://doi.org/10.1097/ACM.0000000000005963

23. Johnston SC. Anticipating and training the physician of the future: the importance of caring in an age of artificial intelligence. *Acad Med*. 2018;93(8):1105-1106. https://doi.org/10.1097/ACM.0000000000002175

24. Han ER, Yeo S, Kim MJ, Lee YH, Park KH, Roh H. Medical education trends for future physicians in the era of advanced technology and artificial intelligence: an integrative review. *BMC Med Educ*. 2019;19(1):460. https://doi.org/10.1186/s12909-019-1891-5

25. Alrassi J, Katsufrakis PJ, Chandran L. Technology can augment, but not replace, critical human skills needed for patient care. *Acad Med*. 2021;96(1):37-43. https://doi.org/10.1097/ACM.0000000000003733

26. Kirkpatrick D, Kirkpatrick JD. *Evaluating Training Programs: The Four Levels*. 3rd ed. Berrett-Koehler Publishers; 2006.

27. Wu MJ, Zhao K, Fils-Aime F. Response rates of online surveys in published research: a meta-analysis. *Comput Hum Behav Rep*. 2022;7:100206. https://doi.org/10.1016/j.chbr.2022.100206

Received: February 5, 2026
Accepted: May 8, 2026
Published: June 19, 2026

Copyright © 2026 Fernandes et al. This is an open-access publication distributed under the terms of the Creative Commons Attribution license.
